# Precision livestock farming: from where we came and where we go

**DOI:** 10.5713/ab.260110

**Published:** 2026-03-11

**Authors:** Daniel Berckmans

**Affiliations:** 1 Department of BioSystems and Soil Sciences, University of Tennessee, Knoxville, TN, USA

**Keywords:** Continuous Monitoring, Different Animal Species, Precision Livestock Farming, Process Efficiency

## Abstract

This paper describes how animal management was done in the past and how we evolved to continuous animal monitoring by using technology. Based upon peer-reviewed literature, we show several technologies (cameras, microphones, sensors) developed and used for monitoring and managing indoor farm animals applied to different animal species: broilers, pigs, dairy cows, and horses. The main idea in developing precision livestock farming (PLF) technology is continuous 24/7 monitoring to generate objective data for farmers, veterinarians, and other stakeholders (pharma, feed company, equipment, climate control, etc.). Development started in laboratory settings and evolved to data collection in commercial farms. This paper shows that PLF monitoring allows us to measure objective data in each term of the fundamental process equation in producing animal products: the transfer from feed energy into animal product (meat, milk, eggs, fiber, and reproduction). The accuracies obtained are impressive in several examples. Regarding where to go with PLF, we show the great importance of animal welfare in the efficiency of the production process. Improving process efficiency is key in avoiding the need for even more farm animals to be slaughtered every year to fulfill the increasing worldwide demand for animal products. Field implementation of PLF technology for continuous objective measurements on indoor farm animals, to improve the efficiency of the production process, is an important contribution to feeding the worldwide increasing demand for animal products without a high increase of the number of farm animals.

## INTRODUCTION

The global production of animal-derived foods is encountering profound and multifaceted challenges. Current estimates indicate that more than 70 billion terrestrial animals are slaughtered annually for human consumption, and projections suggest that global demand for animal products may increase by up to 65% by 2050. Given the constraints already observed within the sector, a proportional expansion of livestock populations is neither feasible nor sustainable.

A primary challenge concerns the interdependence of animal health, human health, and environmental health, as emphasized in the One Health framework. More than 65% of emerging infectious diseases in humans originate from zoonotic pathogens, underscoring the public-health implications of intensive animal production systems. In addition, the environmental footprint of the livestock sector—including greenhouse gas emissions, land degradation, water use, and biodiversity loss—exceeds ecologically sustainable thresholds. Another critical yet often underappreciated issue is the relationship between animal welfare and production efficiency. Physiological stress and suboptimal living conditions impair animals’ capacity to convert feed energy into edible products, thereby reducing productivity and increasing resource use with corresponding environmental impact. Climate change is expected to exacerbate these inefficiencies, as animal production goes together with metabolic heat production and heat stress directly compromises these processes and animal performance in many regions. The sector also carries significant social implications. Farmers face increasing economic pressure to produce high-quality animal products at low consumer prices, contributing to farmer’s mental-health challenges and reduced social resilience in rural communities. Overall, nine of the seventeen United Nations Sustainable Development Goals, adopted by 193 member states in 2015, are linked to livestock production systems. This underscores the central role of the animal-production sector in achieving global sustainability, public-health, and socio-economic objectives.

## WHERE DID WE COME FROM

Historically, the monitoring of farm animals relied primarily on farmers who managed relatively small herds and spent substantial time observing individual animals. As farm sizes have decreased in number but increased dramatically in animal population, this traditional approach to herd management has become untenable. The high stocking density on modern farms limits the ability of farmers and caretakers to conduct sufficient individual animal assessments, resulting in health and welfare problems that often remain undetected. The research in the Welfare Quality protocol [1] provides a valuable gold standard for evaluating animal welfare at the farm level. However, these assessments are very periodic, labor-intensive and expensive, making them unsuitable for continuous monitoring or for supporting daily management decisions.

Advanced monitoring technologies have been used in scientific research for decades—for example, early work [2–4] demonstrated the potential of using technology in understanding animal physiologically. Precision livestock farming (PLF) builds on this foundation by aiming for continuous, automated, real-time monitoring of animals on farms in the field. Depending on the sensor type, PLF systems may record data at extremely high temporal resolution, such as one measurement per second, 23 video frames per second, 20,000 audio samples per second, or continuous streams of accelerometer or heart-rate data.

PLF can be defined as a management tool that uses modern technologies to collect objective data continuously and automatically on production, reproduction, health, welfare, and environmental parameters in farm animals [5]. The term “tool” emphasizes that these technologies are intended to support—not replace—the expertise of farmers, caretakers, veterinarians, and other stakeholders. The integration of sensors such as cameras, microphones, and wearable devices enables objective, data-driven insights that enhance decision-making while maintaining human oversight.

The concept of employing advanced technologies—such as cameras, microphones, and environmental or physiological sensors—not only for experimental research but also for continuous, real-time monitoring of livestock at the farm level was initially met with considerable skepticism and doubts, as noted by Wathes et al [6]. Early concerns centered on feasibility of field implementation, technology and data reliability, and the practical value of continuous measurements in commercial production environments. In 2003, the first biennial European Conference on PLF was established, reaching its 12th edition in 2026. Since then, complementary PLF conferences have emerged in the United States and Asia, reflecting the global expansion of the field. Research presented at these meetings initially focused on the development of enabling technologies, including sensor hardware, data-transfer systems, and analytical algorithms capable of extracting biologically meaningful information from continuous data streams.

As demonstrated in this paper, many of the early technical challenges have now been resolved. Consequently, scientific attention has shifted toward identifying which measurable variables most effectively contribute to improved production efficiency under commercial conditions. This shift has broadened the disciplinary landscape of PLF research, attracting increasing participation from animal scientists, veterinarians, nutritionists, feed specialists, and climate-control experts. This interdisciplinary evolution is particularly valuable, as it reinforces the central principle that the animal itself remains the core component of any livestock production system.

## WHERE DO WE GO WITH PRECISION LIVESTOCK FARMING

Global population growth, combined with rising household incomes in many regions, has led to a sustained increase in the consumption of animal-derived foods. As purchasing power improves, dietary patterns typically shift toward higher intakes of meat, milk, and eggs, resulting in a worldwide demand for animal products that continues to expand. According to Rosling et al [7], this trend is expected to persist until at least 2050, when demographic transitions associated with higher income levels—such as reduced family size—may begin to moderate the rate of demand growth.

**Figure 1:**
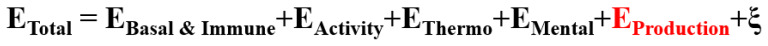
Feed intake provides the total energy (E_Total_), which is allocated to basal metabolism and immune activity (E_Basal & Immune_), physical activity (E_Activity_), thermoregulation (E_Thermo_), and welfare-related processes (E_Mental_). Only the remaining fraction of E_Total_ is used for the formation of animal products (E_Production_) such as meat, milk, eggs, reproduction, and fiber. Production efficiency increases as a larger proportion of energy is allocated to the formation of these animal products.

Meeting global nutritional needs without further expanding livestock populations requires substantial improvements in production efficiency. This challenge underscores the importance of understanding the fundamental biological processes underlying all livestock systems: the conversion of dietary energy into animal products (Figure 1). Feed intake is first allocated to basal metabolic functions necessary to maintain organ activity and homeostasis. Only after these basic maintenance requirements are met can remaining nutrients be directed toward other components in the core equation. Another essential component of the animal’s energy budget is the immune system, which requires substantial metabolic resources to maintain readiness and respond to pathogenic challenges. Thermoregulation represents a further major energy-consuming process, as animals must maintain a stable core body temperature despite fluctuations in environmental conditions such as ambient temperature, humidity, air velocity, and solar or thermal radiation. Animal activity also contributes to overall energy expenditure, ranging from subtle movements (e.g., ear or tail motion) to more intensive behaviors such as standing, walking, playing, or aggressive interactions. In addition, cognitive and emotional states—central to the concept of animal welfare—constitute a further dimension of biological functioning that influences both behavior and physiological responses. Finally, the production outputs of the animal—meat, milk, eggs, reproductive performance, and fiber—represent the portion of energy that remains available after all maintenance and regulatory processes have been met. Each of these components draws from the same fundamental energy pool derived from feed intake.

PLF provides tools for monitoring and managing these processes, but its more advanced objective is to quantify and regulate each component of this fundamental biological equation. By doing so, PLF supports a more precise understanding of how animals allocate energy and enables more efficient and welfare-oriented production systems and management of farm animals.

## CONTINUOUS MONITORING OF FEED INTAKE

**Figure 2:**
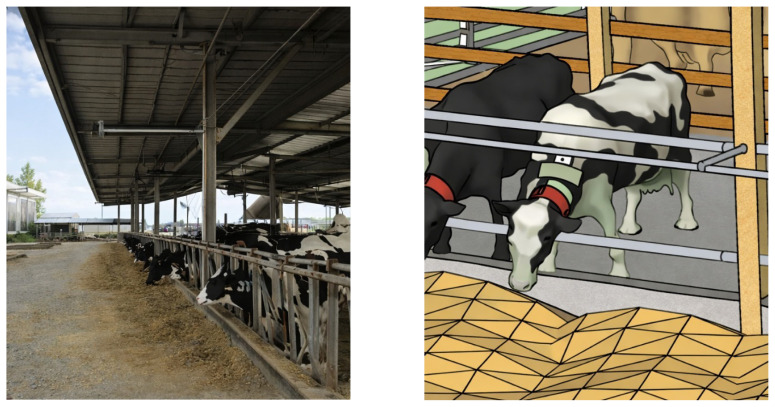
Vision-based precision livestock farming technology for continuous, on-farm monitoring of individual feed intake in dairy cows based on an RGB-D camera system and deep learning algorithms. Over a 36-h period, the system correctly identified 93.65% of the cows and accurately measured the amount of feed consumed per meal, which ranged from 0 to 8 kg, with mean absolute and mean square errors of 0.127 kg and 0.034 kg, respectively [8].

An example is the PLF technology developed to monitor feed intake by dairy cows on farm via image analysis (Figure 2; [8]). Images of cows eating over a period of 36 h provided the test data for cow identification showing that the system was able to accurately identify 93.65% of the cows. The amount of feed consumed which ranged from 0 to 8 kg per meal, was measured with mean absolute and square errors of 0.127 kg, and 0.034 kg, respectively. The accuracy of the technique is exceptional good seen the total amount of feed intake by dairy cows [8]. For cows in the pasture ingestive events, feeding time and feed intake monitoring has been reported in literature. Another example of PLF technology continuously monitoring feed intake is by sound analysis of broiler peckings collected via a contact-microphone attached in the feeder pan. A feeder pan was positioned on an accurate weighing scale and a collection of spoiled feed as gold standard for the amount eaten by the bird. A PLF algorithm detects the feed intake from the pecking sounds with a maximal error of 0.2 g over a 30 min period [9]. Table 1 shows that on a total of 3,444 peckings the algorithm detected 7.5% more peckings than detected manually from video analyses. Table 2 shows that for 300 min of monitoring sound with over 25,000 peckings the amount of feed disappearing, as measured by the weighing scale, showed an average feed intake per pecking of 0.025 g and an average loss of feed of only 0.98% per experiment in 12 experiments. In 2025, 17 yr after this research, finally a project is running at the University of Tennessee (Knoxville, TN, USA) developing a commercial product for broiler houses using this technology.

## CONTINUOUS MONITORING OF DISEASES AND INFECTION

Continuous animal health status has long been recognized as essential like in relation to intestinal integrity in poultry populations [10]. A fundamental priority in both human and veterinary physiology is ensuring that the immune system receives adequate metabolic energy. When energy is diverted toward competing physiological demands—such as thermoregulation, and locomotion, responses to environmental and to mental stressors like the lack of animal welfare—the immune system may experience an energy deficit. This deficit can compromise immune competence and increase susceptibility to infectious diseases. Reducing infection pressure and minimizing the need for antimicrobial interventions not only improve the efficiency with which dietary energy is converted into animal-derived food products but also contribute to lowering the environmental footprint associated with pharmaceutical use [11].

**Figure 3:**
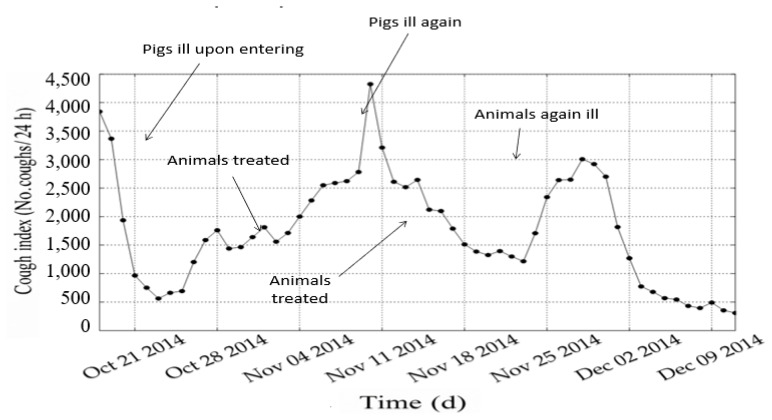
Results from SoundTalks monitor for respiratory diseases for fattening pigs versus late treatment by farmer and veterinarian.

**Figure 4:**
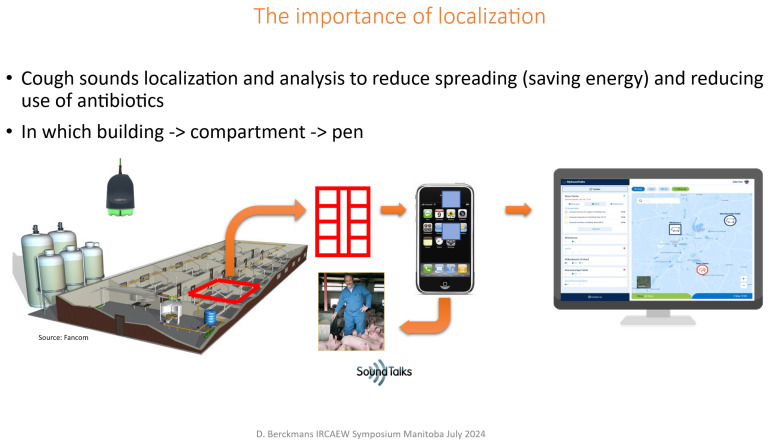
Sound monitor detects in which building, in which compartment and in which pen a respiratory disease is detected and farmer can inform the vet and the regional animal services if required.

Between 2002 and 2008, researchers investigated automated, real-time detection of pig cough sounds as an indicator of respiratory disease, demonstrating the feasibility of acoustic monitoring for early disease recognition [12,13]. Building on this foundational work, SoundTalks established a company in 2011 to develop a commercial PLF system capable of continuously monitoring respiratory health in commercial pig facilities. The system analyzes cough patterns, identifies deviations associated with respiratory infections, and generates alerts both at the individual animal level and for the farm management [14]. Figure 3 presents the outcomes of a blind assessment in which the farmer did not have access to the alerts generated by the PLF monitor. This setup was used to evaluate how rapidly the farmer could identify a disease outbreak compared with the automated pig-sound monitoring system. The fattening pigs already exhibited clinical illness at the start of the fattening period, and the farmer-initiated treatment only after 10 d. A second infection was detected by the monitoring system and subsequently treated 5 d later, whereas a third infection remained entirely unnoticed and untreated by the farmer. During each infection event, the animals showed a marked reduction in feed intake, resulting in substantial production losses across the fattening cycle. The monitoring system issues an alert and can localize the affected animal(s) with spatial precision—indicating the building, the specific compartment, and the exact pen where attention is required. In cases of severe or widespread infection, regional animal health authorities can also identify emerging disease patterns across farms collecting these data (Figure 4).

## CONTINUOUS MONITORING AND REDUCTIOPN OF ENERGY CONSUMPTION IN ANIMAL ACTIVITIES

Physical activity, including walking and playing behavior, is essential for maintaining health and welfare in pigs. Social groups naturally establish dominance hierarchies, which can lead to occasional aggressive interactions. However, intense or prolonged fighting is undesirable, as it increases metabolic expenditure, elevates stress levels and can reduce carcass quality. To address this, a fighting-detection algorithm was developed and validated using top-view video recordings of pens containing groups of 10 fattening pigs [15]. The system achieved an accuracy of 89% in identifying aggressive events. In a complementary behavioral intervention study, piglets were conditioned before weaning to associate an acoustic signal with the delivery of a sweet reward. After weaning, the same acoustic signal was used during aggressive encounters. Remarkably, 74.9% of aggressive actions were interrupted solely by the sound cue, even though no reward was provided during the post-weaning phase [16].

Using top-view camera images offers a major advantage in automated behavior analysis because the background remains stable, allowing animals to be distinguished more easily from their surroundings with high reliability. This approach also demonstrates that features traditionally extracted from side-view images can be successfully adapted for top-view analysis. A similar principle has been applied to another energy-demanding activity: locomotion in animals with lameness. In a dataset comprising 223 videos from 90 dairy cows, image-based analysis correctly classified 91% of lame individuals [17]. Lameness detection was originally performed using side-view images, where the curvature of the back (back arch) emerged as the most informative feature among 20 evaluated parameters, including step overlap, stride length, and hoof contact angle. When a cow experiences pain or dysfunction in one limb, it reduces weight-bearing on that leg. This compensation requires increased activation of the back musculature, resulting in a measurable change from the top-view in the animal’s back arch [18]. This work also demonstrated that classification performance improved when individualized models were used instead of a single population-level model. When applying a group-based threshold, 76% of lame cows were correctly classified, with a true positive rate of 83% and a false positive rate of 22%. In contrast, using individualized thresholds increased both overall accuracy and the true positive rate by ten percentage points, reaching 91%, while the false positive rate decreased by four percentage points to 6%. These findings reinforce a fundamental principle in PLF: animals differ substantially at the individual level. While group averages are statistically useful for comparing treatment groups, they provide limited insight into the condition or behavior of individual animals within those groups [18].

## BODY ENERGY USED FOR THERMAL CONTROL

**Figure 5:**
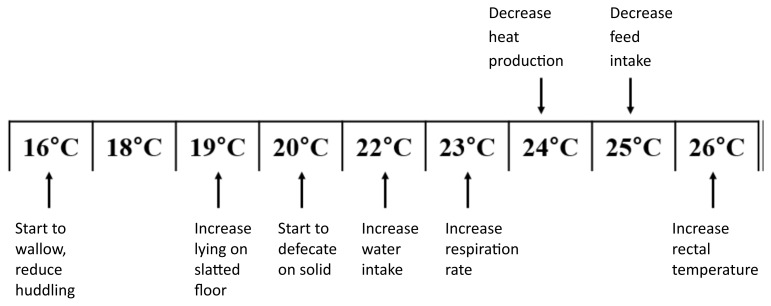
Effect of environmental temperature on pig responses scored in commercial pig houses. Data from Huynh [23].

It is well established from decades of research that the microclimate surrounding an animal—characterized by temperature, humidity, air velocity, radiation, and concentrations of gases and dust—has a substantial impact on production efficiency, including growth rate and feed conversion. Laboratory studies have long demonstrated that maintaining thermal homeostasis under suboptimal climatic conditions requires considerable metabolic energy [19–22]. The influence of ambient temperature on the growth and development of fattening pigs has also been quantified under commercial conditions. Measurements in operational pig facilities confirmed that deviations from the thermal comfort zone significantly reduce growth performance in fattening pigs (Figure 5; [23]). Inside climate control in commercial farm animal houses is used for long and 20 yr ago several companies providing equipment for livestock houses had experts on the road helping farmers in setting the electronic climate controllers. Today, companies save costs and have reduced the manpower supporting farmers in the use of complex digital climate controllers. Besides the veterinarian, there is no regular expert visit in farms, unless the farmer is asking and paying for it. Nobody is familiar with the complex digital control software, and nobody is busy with it. The result is that the control of the climate is in many cases worse than it used to be 20 yr ago. This is also a risk when AI climate controllers will be introduced in the field.

## ENERGY LOSSES DUE TO LACK OF ANIMAL WELFARE

**Figure 6:**
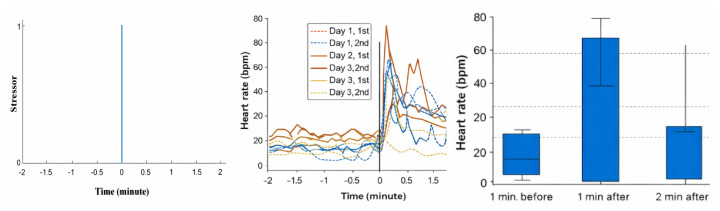
Lack of animal welfare is detected via de mental component in heart rate. Data from Joosen et al [25].

**Figure 7:**
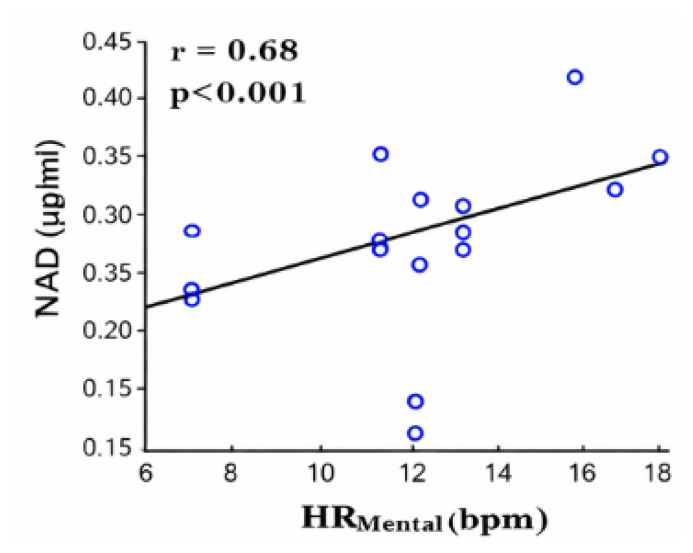
The correlation between the real-time detected mental component of heart rate (HR_Mental_) and the concentration of the blood hormone noradrenaline (NAD) in stressful event for pigs.

When considering the use of feed energy in the core production equation (Figure 1), it is important to recognize that not only physiological processes, but also mental states influence the animal’s overall energy budget. Modern production systems often impose welfare challenges—such as limited space, barren environments for cognitively complex species like pigs, insufficient enrichment, and social aggression—that can induce frustration and stress. As documented in the literature, such negative mental states require metabolic energy, diverting resources away from growth and other productive functions. A real-time algorithm originally developed for humans using wearables [24], has been adapted to quantify the mental component in animals. This algorithm was tested in pigs exposed to both negative stressors and positive play stimuli [25]. Heart rate and physical activity were continuously recorded every second, and the algorithm decomposed heart rate into basal, physical, and mental components. The results show that when the animals were exposed to an acute auditory stressor, the real-time algorithm detected the onset of stress immediately (Figure 6). The experiments also demonstrated that the pigs’ responses varied across repeated trials on different days, as reflected in the time-varying patterns in Figure 6. Although statistical analysis did not reveal significant differences in the mental component of heart rate before, during, or after the stress event, the algorithm consistently identified the precise moment at which the stressor occurred. Moreover, Figure 6 shows that a statistical analysis does not show any significant difference in the mental heart rate component before, during, and after the experiment. But the figure also shows that the algorithm clearly detects the presence of the stressor at the right moment. Furthermore, the relationship between the algorithm’s mental-activity output and circulating noradrenaline concentrations is illustrated in Figure 7 (r = 0.68; p<0.001). The data points appear scattered, largely because noradrenaline is released rapidly and transiently, making it difficult to obtain blood samples at the exact physiological peak. Nevertheless, the correlation confirms that mental energy expenditure during stress can be quantified, and its association with noradrenaline has been demonstrated [25]. Although the technology for detecting this mental component is available, a robust, animal-friendly commercial heart-rate sensor suitable for continuous use in livestock is not yet on the market.

## ENERGY GOING INTO THE ANIMAL PRODUCT

**Figure 8:**
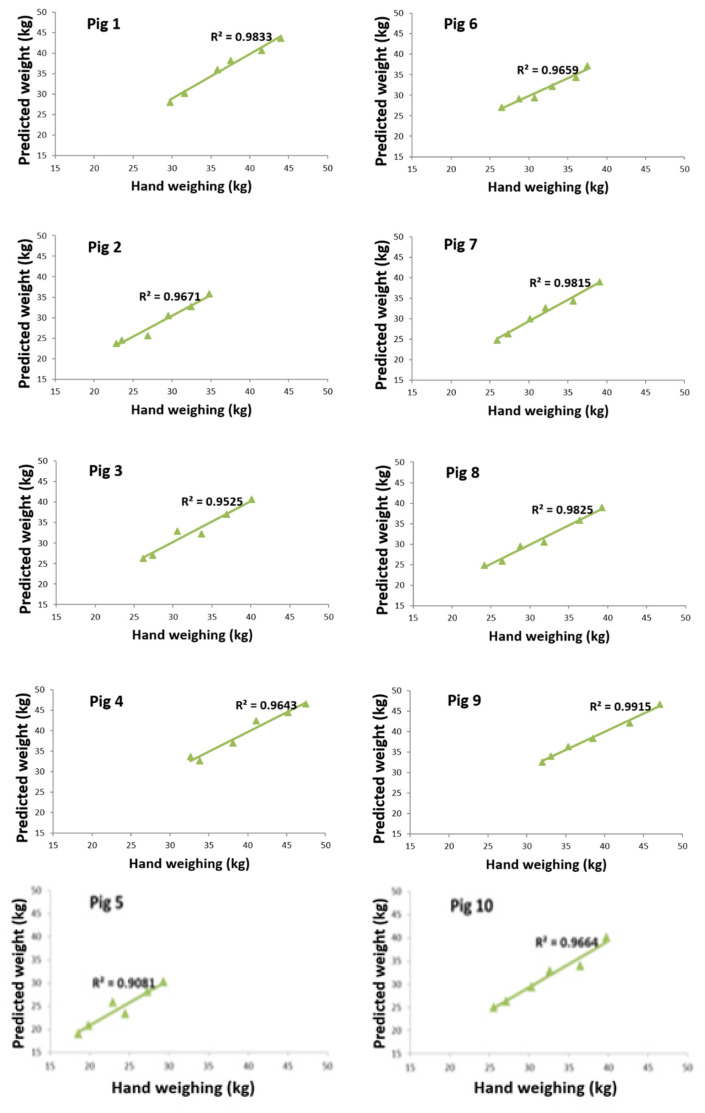
The high correlations between individual weight estimated from computer vision from a top-view camera and the hand weighing of pigs.

When feed energy remains after covering all the higher described components of the production energy equation, the surplus becomes available for the formation of animal products. The challenge for modern livestock systems is to increase the efficiency of this process: producing more output with less feed input while minimizing energy losses associated with compromised welfare, immune activation, and non-productive physical activities such as aggression and intense social fighting. Performance indicators such as feed conversion ratio, growth rate, mortality, and disease-related costs have long been used to evaluate farm animal productivity. As illustrated in Figure 8, the body weight of groups of ten pigs can be estimated with high accuracy using a simple 2D top-view camera system, providing farmers with valuable information for management decisions [26]. Although the maximum prediction error of 0.84 kg is still too high for use in young piglets, the correlation between camera-based weight estimation and reference hand-weighing for individual fattening pigs reaches approximately 96%, demonstrating strong potential for practical on-farm application. Current automated pig-weighing technologies represent an important step toward this goal. State-of-the-art systems using 3D camera technology combined with artificial intelligence achieve weight-estimation errors between 2% and 3.6%. These non-contact solutions—such as eYeGrow (Fancom)—enable continuous, 24/7, stress-free monitoring of pig weight and growth, providing farmers with accurate, real-time data to support more efficient management decisions.

## MODEL BASED PREDICTIVE CONTROL

The preceding sections demonstrate that each component of the core energy equation in animal production (Figure 1) can already be monitored continuously and with acceptable accuracy using PLF technologies. When a process variable can be measured 24/7 with sufficient precision, it becomes feasible to model its dynamic behavior. Once a model can predict how an output variable like weight gain responds to changes in a controllable input like supply, the system can be optimized—minimizing energy losses, improving accuracy, and enhancing overall production efficiency. This principle of predictive control is widely applied in many technological domains and, increasingly, in modern livestock management as well. Humans intuitively apply predictive control in everyday life. For example, when riding a bicycle or driving a car, the brain relies on an internal model that predicts how the vehicle will respond to steering inputs. When this internal model becomes inaccurate, such as on an icy road—control deteriorates. The same principle underlies every efficient process-control system: accurate prediction enables optimal adjustment. This concept is particularly important in animal production, where biological systems are inherently time-varying. Model-based predictive control has already been demonstrated in broiler production, where dynamic models of growth have been used to optimize environmental and management inputs. In 12 experiments involving 2,900 broilers, daily body weight and feed intake were recorded using an automated weighing scale [27] and precise measurement of feed supply. These data enabled the development of a daily predictive model describing the birds’ weight response to feed allocation. Such a model forms the basis for calculating the exact amount of feed required to achieve a predefined growth trajectory over time.

**Figure 9:**
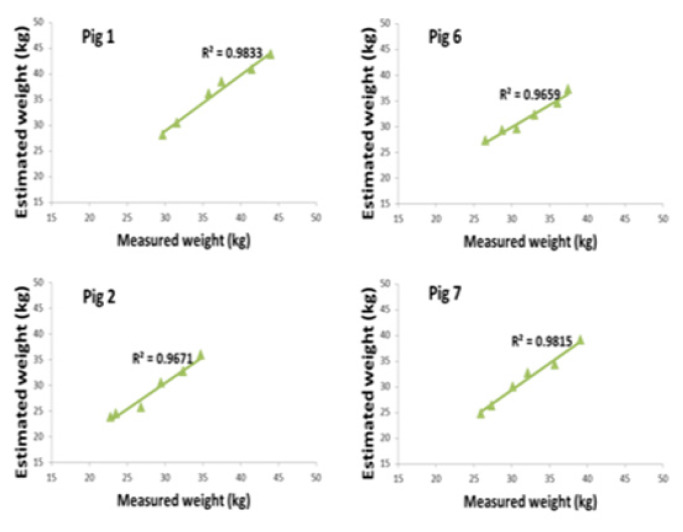
Precision livestock farming (PLF)-model-based-predictive controls results in a different growth trajectory with reduced mortality and lameness. The PLF control results in reduction of mortality of 4% (12 Exp.; 2,900 animals each).

Literature describes this approach in detail, measuring the control input (feed supply) and the process output (body weight) on a daily basis [28,29]. In their study, the control group received *ad libitum* feeding, whereas the treatment group was provided with a controlled feed allowance designed to follow a specified growth curve. The underlying rationale was to slow early growth to allow adequate skeletal development before rapid muscle deposition occurred, thereby reducing the risk of lameness and mobility-related feeding problems in heavier birds. The mean relative error between the predefined target growth trajectories and the realized trajectories ranged from 3.7% to 6.0% (Figure 9). In terms of flock uniformity, the controlled-feeding group showed a higher uniformity index than the ad libitum group in Trial 2 (56.3% vs. 50.1%), although the opposite was observed in Trial 1 (47.0% vs. 55.0%). Mortality was substantially reduced in the controlled-feeding group, decreasing from 3.1% in the *ad libitum* group to 1.2% in the treatment group. Another notable example is the training of sport horses that was much more efficient when using model predictive control [30].

## CONCLUSION

Farmers in earlier generations typically managed only a small number of animals, relying entirely on direct audio-visual observation. Although most farms worldwide are still relatively small, the majority of global animal-product volume now comes from very large operations, some housing more than 120,000 dairy cows on a single site. Research across a wide range of livestock species has consistently demonstrated that PLF technologies are capable of providing reliable, continuous 24/7 monitoring in lab-controlled environments and more and more also under commercial conditions.

Since the earliest attempts to use monitoring technologies for scientific purposes, there has been considerable skepticism about whether such systems could ever be deployed effectively in large-scale, real-world livestock environments. However, the accumulated evidence now clearly shows that PLF systems can operate robustly in commercial settings, offering farmers continuous, objective, and actionable information about their animals.

A major challenge today is that many researchers continue to focus on developing new methods, often more complex, more expensive, and even further removed from practical implementation—rather than advancing technologies that have already proven their value over the past two decades. Too few research groups take the crucial step of translating their findings into usable products for the field. What is urgently needed is the transfer of validated research into commercial solutions that demonstrate a clear return on investment, rather than producing yet another method that achieves results already demonstrated multiple times. Large-scale farms, which may house tens of thousands of animals, cannot rely on manual observation and therefore represent the strongest drivers for PLF product development. However, early-stage production of new technologies inevitably involves low manufacturing volumes, which keeps costs high. Only when these systems are adopted more widely—and produced at scale—will costs decrease and accessibility improve. Wider adoption of PLF technologies in large-scale farms will increase production volumes of PLF products and, consequently, reduce their cost. This reduction is essential to make PLF solutions accessible to small farms and family-run operations, which still represent the majority of livestock producers worldwide. Research and industry should therefore also prioritize the development of simple, affordable PLF tools tailored to the needs and constraints of these smaller farms, ensuring that technological progress benefits the full spectrum of animal production systems.

## Figures and Tables

**Table 1:** Results detecting number of peckings for feed intake monitoring using sound analysis of pecking sounds via microphone in a feeder pan

Data set^1)^	Number of peckings (algorithm)	Number of peckings (video labelling)	Accuracy of algorithm	True positive	False positive
1	113	105	93	105	8
2	99	95	96	95	8
3	109	106	98	106	4
34	107	101	94	101	3
35	98	91	93	91	6
36	95	88	92	88	7
Average	103	96	93	96	7

1) Individual-level data for data sets 4 to 33 were not reported; only mean values were reported in the original publication [9].

**Table 2:** Accuracies of feed intake (FI) monitoring using sound analysis of pecking sounds via microphone in a feeder pan

Chickens^1)^	Exp.	Min	No. of peckings per Exp.	FI per Exp. (g)	Feed loss per Exp. (g)	FI per Exp. (g)	FI per pecking (g)	FI per pecking (mean±SD)	Feed loss per Exp.
1	1	13.00	1,193	28.63	0.325	28.31	0.024	0.025±0.0015	1.14
	2	12.00	759	18.98	0.198	18.78	0.025		1.04
	3	10.30	895	24.17	0.222	23.94	0.027		0.92
2	1	15.00	1,250	32.50	0.236	32.26	0.026	0.025±0.0012	0.73
	2	13.50	1,283	30.79	0.365	30.43	0.024		1.19
	3	15.00	1,460	35.04	0.348	34.69	0.024		0.99
3	1	7.04	651	16.28	0.168	16.11	0.025	0.025±0.0006	1.03
	2	4.35	468	12.17	0.111	12.06	0.026		0.91
	3	7.26	533	13.33	0.124	13.20	0.025		0.93
12	1	6.54	583	13.99	0.145	13.85	0.024	0.025±0.0015	1.04
	2	7.43	654	16.35	0.165	16.19	0.025		1.01
	3	6.65	573	15.47	0.155	15.32	0.027		1.00
Average		8.34	702	17.59	0.173	17.42	0.025	0.025±0.0011	0.98

1) Individual-level data for chickens 4 to 11 were not reported; only mean values were reported in the original publication [9].

SD, standard deviation.

## Data Availability

Upon reasonable request, datasets of this study can be available from the corresponding author.

## References

[1] Blokhuis HJ Ekkel ED Korte SM Hopster H van Reenen CG Farm animal welfare research in interaction with society Vet Q 2000 22 217 22 10.1080/01652176.2000.9695062 11087134

[2] Pettibone CA Scott NR Relationship of temperatures in the cervical blood vessels to brain temperatures in chickens Trans ASAE 1976 19 0736 42 10.13031/2013.36107

[3] Rothschild MF Bodoh GW Pearson RE Miller RH Sources of variation in quarter milk flow measures J Dairy Sci 1980 63 1138 44 10.3168/jds.S0022-0302(80)83059-1

[4] Kettlewell PJ Mitchell MA Meeks IR An implantable radio-telemetry system for remote monitoring of heart rate and deep body temperature in poultry Comput Electron Agric 1997 17 161 75 10.1016/S0168-1699(96)01302-6

[5] Berckmans D Automatic on-line monitoring of animals by precision livestock farming Geers R Madec F Livestock production and society Brill 2006 287 94

[6] Wathes CM Kristensen HH Aerts JM Berckmans D Is precision livestock farming an engineer’s daydream or nightmare, an animal’s friend or foe, and a farmer’s panacea or pitfall? Cox S Precision livestock farming ‘05 Brill 2005 33 46

[7] Rosling H Rönnlund AR Rosling O Factfulness: ten reasons we’re wrong about the world and why things are better than you think Flatiron Books 2018

[8] Bezen R Edan Y Halachmi I Computer vision system for measuring individual cow feed intake using RGB-D camera and deep learning algorithms Comput Electron Agric 2020 172 105345 10.1016/j.compag.2020.105345

[9] Aydin A Bahr C Viazzi S Exadaktylos V Buyse J Berckmans D A novel method to automatically measure the feed intake of broiler chickens by sound technology Comput Electron Agric 2014 101 17 23 10.1016/j.compag.2013.11.012

[10] Ducatelle R Goossens E De Meyer F Biomarkers for monitoring intestinal health in poultry: present status and future perspectives Vet Res 2018 49 43 10.1186/s13567-018-0538-6 29739469 PMC5941335

[11] Tullo E Finzi A Guarino M Review: environmental impact of livestock farming and precision livestock farming as a mitigation strategy Sci Total Environ 2019 650 2751 60 10.1016/j.scitotenv.2018.10.018 30373053

[12] Van Hirtum A Berckmans D Ruano AE Ruano MG Fleming PJ Intelligent free field cough sound recognition Proceedings of the International Conference on Intelligent Control Systems and Signal Processing (ICONS’03) 2003 Apr 8–11 Faro, Portugal Pergamon 2003 453 8

[13] Exadaktylos V Silva M Ferrari S Time-series analysis for online recognition and localization of sick pig (Sus scrofa) cough sounds J Acoust Soc Am 2008 124 3803 9 10.1121/1.2998780 19206806

[14] Vranken E Berckmans D Precision livestock farming for pigs Anim Front 2017 7 32 7 10.2527/af.2017.0106

[15] Viazzi S Ismayilova G Oczak M Image feature extraction for classification of aggressive interactions among pigs Comput Electron Agric 2014 104 57 62 10.1016/j.compag.2014.03.010

[16] Ismayilova G Sonoda L Fels M Acoustic-reward learning as a method to reduce the incidence of aggressive and abnormal behaviours among newly mixed piglets Anim Prod Sci 2014 54 1084 90 10.1071/AN13202

[17] Viazzi S Bahr C Schlageter-Tello A Analysis of individual classification of lameness using automatic measurement of back posture in dairy cattle J Dairy Sci 2013 96 257 66 10.3168/jds.2012-5806 23164234

[18] Viazzi S Bahr C Van Hertem T Comparison of a three-dimensional and two-dimensional camera system for automated measurement of back posture in dairy cows Comput Electron Agric 2014 100 139 47 10.1016/j.compag.2013.11.005

[19] Morrison SR Heitman H Jr Bond TE Effect of humidity on swine at temperatures above optimum Int J Biometeorol 1969 13 135 9 10.1007/BF01552734 5379092

[20] Sugahaea M Baker DH Harmon BG Jensen AH Effect of ambient temperature on performance and carcass development in young swine J Anim Sci 1970 31 59 62 10.2527/jas1970.31159x

[21] Holmes CW McLean NR The effect of low ambient temperatures on the energy metabolism of sows Anim Sci 1974 19 1 12 10.1017/S0003356100022534

[22] Dauncey ML Ingram DL Walters DE Legge KF Evaluation of the effects of environmental temperature and nutrition on growth and development J Agric Sci 1983 101 291 9 10.1017/S0021859600037588

[23] Huynh TTT Heat stress in growing pigs [dissertation] Wageningen University & Research 2005

[24] BioRICS BioRICS reveals what your body already knew [Internet] BioRICS 2026 [cited 2026 Feb 1]. Available from: https://www.biorics.com

[25] Joosen P Norton T Marchant-Ford J Berckmans D Animal welfare monitoring by real-time physiological signals Prec Livest Farm 2019 19 337 44

[26] Kashiha M Bahr C Ott S Automatic weight estimation of individual pigs using image analysis Comput Electron Agric 2014 107 38 44 10.1016/j.compag.2014.06.003

[27] Vranken E Chedad A Aerts JM Berckmans D Improving the accuracy of automatic broiler weighing by image analysis Cox S Precision livestock farming ‘05 Brill 2005 265 71

[28] Berckmans D Aerts JM Van Buggenhout S Controlling growth of broiler chickens on-line, based on a compact predictive growth model Cox S Precision livestock farming Brill 2003 27 32

[29] Aerts JM Van Buggenhout S Vranken E Active control of the growth trajectory of broiler chickens based on online animal responses Poult Sci 2003 82 1853 62 10.1093/ps/82.12.1853 14717542

[30] Aerts JM Gebruers F VanCamp E Berckmans D Controlling horse heart rate as a basis for training improvement Comput Electron Agric 2008 64 78 84 10.1016/j.compag.2008.05.001

